# Recurrent hemorrhagic shock from hemorrhagic cystitis due to neurogenic bladder

**DOI:** 10.1002/iju5.12354

**Published:** 2021-08-12

**Authors:** Teruki Isobe, Kenji Matsui, Kunihiro Ishioka, Yasuhiro Mochida, Hidekazu Moriya, Sumi Hidaka, Takayasu Ohtake, Toshiki Etani, Takahiro Yasui, Shuzo Kobayashi

**Affiliations:** ^1^ Kidney Disease and Transplant Center Shonan Kamakura General Hospital Kamakura Kanawaga Japan; ^2^ Department of Nephro‐urology Graduate School of Medical Sciences Nagoya City University Nagoya Japan

**Keywords:** hemorrhagic cystitis, hemorrhagic shock, neurogenic bladder, urinary tract infection

## Abstract

**Introduction:**

Hemorrhagic cystitis is characterized by gross hematuria, with hemorrhagic shock a rare complication. However, to our knowledge, its exact frequency has not been reported.

**Case presentation:**

We report a case of an 86‐year‐old woman who showed repeated hemorrhagic cystitis with massive bleeding and hemorrhagic shock. The hemorrhagic cystitis was supposedly caused by the administration of aspirin and a neurogenic bladder. A urethral catheter was indwelled and hemorrhagic cystitis subsequently ceased.

**Conclusion:**

A review of patients with hemorrhagic cystitis at our hospital showed that only 3.3% experienced hemorrhagic shock. This case was even rarer because the patient experienced recurrent hemorrhagic shocks. A neurogenic bladder, which reduces the bladder’s ability to function as a uroepithelial barrier against recurrent bacterial infections, caused the condition in this case. This report highlights how hemorrhagic cystitis can sometimes cause hemorrhagic shock.

**Abbreviation & Acronym**
ANCA: anti‐neutrophil cytoplasmic antibodies

**Keynote message**

The recurrence of hemorrhagic cystitis caused by a neurogenic bladder leading to hemorrhagic shock is a rare condition.

## Introduction

Hemorrhagic cystitis exhibits diffuse inflammation, resulting in gross bleeding. Its various etiologies include infection, radiation, or chemical exposure, and the presence of a tumor. However, the cause is not identified in most cases.^1^ Hemorrhagic shock refers to an inadequate perfusion of tissues due to severe blood loss. To our knowledge, very few articles exist in the literature on hemorrhagic cystitis resulting in hemorrhagic shock, a subtype of hypovolemic shock, and the frequency of its occurrence has not yet been reported.^2^

We report a case of recurrent hemorrhagic cystitis with massive bleeding and hemorrhagic shock due to a neurogenic bladder.

## Case report

An 86‐year‐old woman experienced recurrent massive hematuria for the past 4 years that used to disappear temporarily with oral antibiotics. The condition had been diagnosed as bacterial cystitis.

On admission day, her hematuria had lasted for 2 days prior despite taking antibiotics. She was transferred to the emergency department of our hospital.

The patient’s medical history includes chronic heart failure, chronic kidney disease, angina pectoris, subarachnoid hemorrhage, uterine prolapse, and lumbar spinal canal stenosis. She also had a history of surgeries for a uterine myoma and femoral neck fracture and medications included aspirin and carvedilol.

**Fig. 1:**
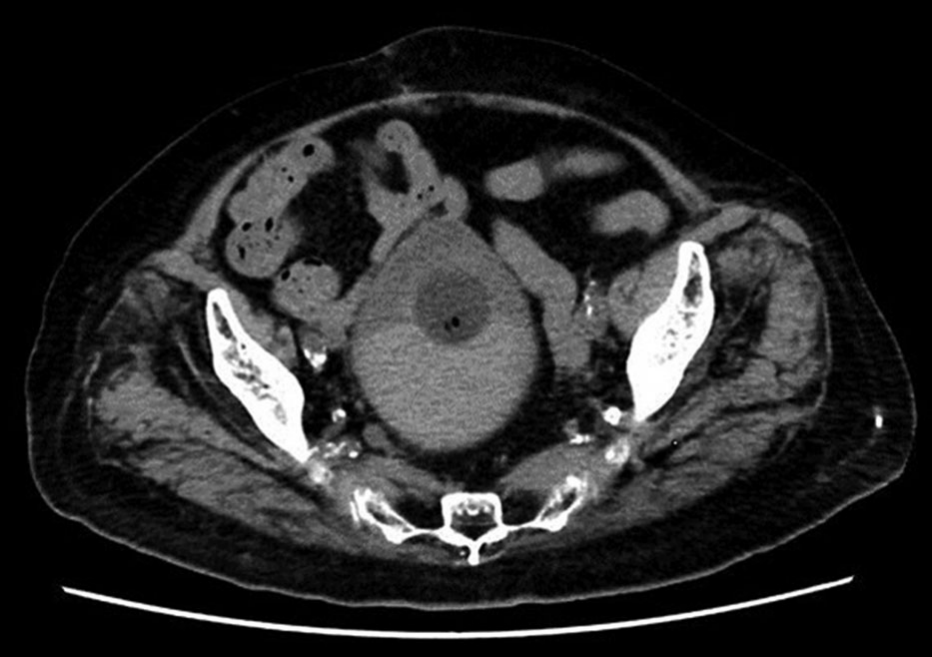
Computed tomography scan showing blood clots in the bladder, but no ascites.

On arrival, she appeared drowsy, and the following were noted: Glasgow Coma Scale, E4V4M6; body temperature, 35.7°C; blood pressure, 90/48 mmHg; and heart rate, 60 bpm. Her conjunctiva was slightly pale, and her extremities were cold. Laboratory findings revealed severe anemia (6.6 g/dL of hemoglobin) and an increased creatinine level (2.53 mg/dL). Her coagulation function was normal. Urinalysis revealed hematuria and pyuria. A urine culture was negative, probably because of the oral antibiotics she had taken before being transferred. On abdominal ultrasonography, the inferior vena cava was observed to be collapsed. A computed tomography scan revealed blood clots in the bladder but no ascites (Fig. 1). Although septic shock from a urinary tract infection was at first suspected, peripheral coldness, the collapse of the inferior vena cava, a negative blood culture, and a low hemoglobin level led to the diagnosis of hemorrhagic cystitis with hemorrhagic shock.

Blood transfusion, continuous bladder irrigation with saline, and a ceftriaxone drip (1 g once a day) were immediately initiated after admission. The hematuria resolved on the second hospital day, and the anemia improved on the third. The patient was discharged 10 days after admission.

**Fig. 2:**
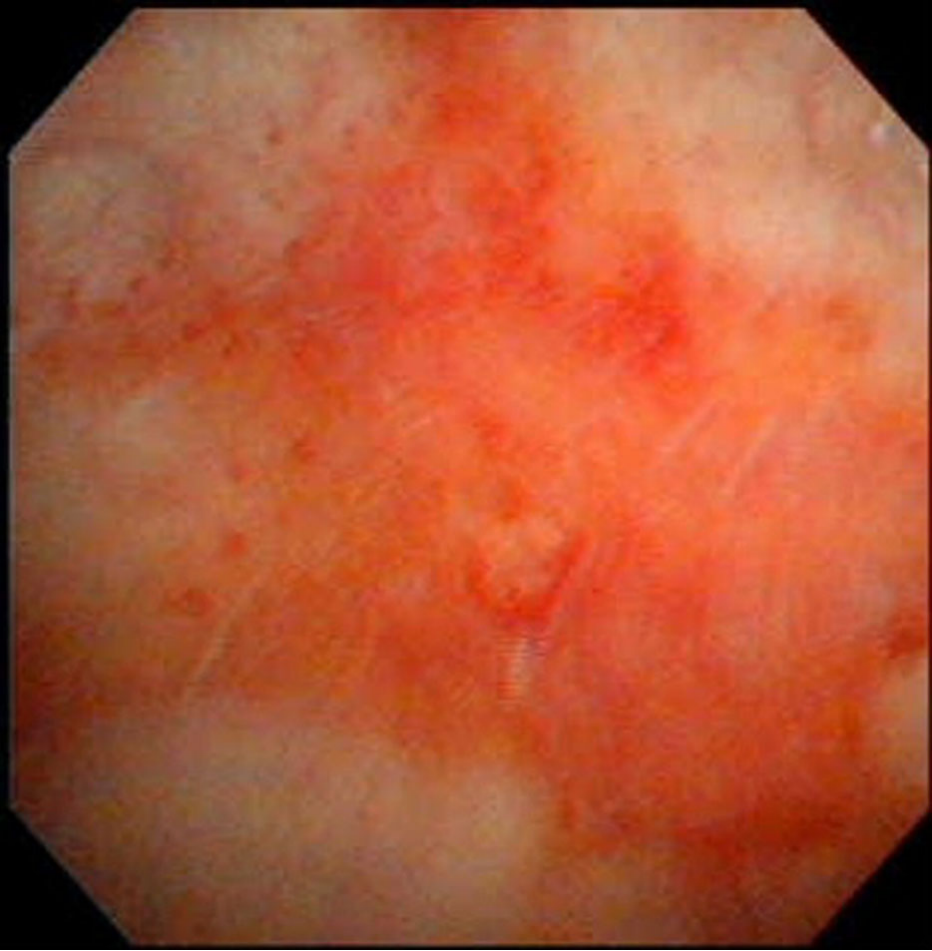
Cystoscopy image on the 25th day of the second admission showing inflammation of the bladder wall, but no malignancy.

Two months later, however, the patient returned to the emergency room complaining of massive gross hematuria. This time, her blood pressure was 62/44 mmHg and heart rate was 62 bpm. Laboratory examination revealed a hemoglobin level of 8.0 g/dL. Noradrenaline was used in addition to the same treatment as had been used in the previous admission. Her blood pressure improved the following day. She underwent cystoscopy, which revealed inflammation of the bladder walls but no tumor (Fig. 2). Tests for anti‐myeloperoxidase ANCA and anti‐proteinase‐3 ANCA, cytomegalovirus, urinary BK virus, and urinary adenovirus were negative. Hematuria ceased on the ninth admission day.

During her hospitalization, the patient experienced frequent episodes of urinary incontinence with residual urine of more than 150 mL. Therefore, she was diagnosed with a neurogenic bladder. Her past medical history of a lumbar spinal canal stenosis and total hysterectomy for a uterine fibroid were suspected as the underlying causes of the neurogenic bladder. A urinary catheter was inserted to stop the recurrence of urinary infection, following which the gross hematuria ceased.

## Discussion

In this report, we describe the case of an 86‐year‐old woman with recurrent hemorrhagic shock resulting from hemorrhagic cystitis, probably due to a neurogenic bladder.

Hemorrhagic cystitis is caused by infectious and noninfectious (radiation, drugs) factors.^2^ However, to our knowledge, its frequency and etiology of hemorrhagic cystitis accompanied by hemorrhagic shock is unknown. As we investigated, few cases of refractory hemorrhagic cystitis with subsequent hemorrhagic shock were reported in the past.^3^, ^4^, ^5^ Chemotherapy and infection mainly cause severe hemorrhagic cystitis. At our hospital, 183 patients were diagnosed with hemorrhagic cystitis between 2009 and 2018. Of these, six patients (3.3%) experienced hemorrhagic shock and only one (0.5%) patient (this case) experienced recurrent hemorrhagic shock. This case was diagnosed with neurologic bladder, but did not receive treatments such as chemotherapy or radiation which commonly recognized as main factor of hemorrhagic shock. This case is rare at this point.

A neurogenic bladder results from a disorder of the nervous system and is accompanied by lower urinary tract dysfunction. In our patient, medical history of a lumbar spinal canal stenosis and total hysterectomy might cause the neurogenic bladder. Generally, patients with a neurogenic bladder experience urinary tract infections 2.5 times per person‐year, which increases with a residual urine volume over 100 mL.^6^ Apart from susceptibility to bacterial infection, a neurogenic bladder is associated with disruption of the bladder epithelial barrier. In a study on the neurogenic bladder, after rats experienced a traumatic spinal cord injury, significant injury in the bladder uroepithelial tight junctions increased their permeability to urine and induced inflammation. This was detected through an increase in urinary protein and hemoglobin levels. These changes were confirmed even during the chronic phase.^7^, ^8^ Therefore, a neurogenic bladder itself can cause hemorrhagic cystitis.

Treatment for a neurogenic bladder includes intermittent catheterization, medication, and surgery.^9^ In this case, the patient could not be provided with intermittent catheterization or be operated on, and a cholinergic drug was not effective. We inserted an indwelling urethral catheter, and hematuria did not recur thereafter.

The relationship between the presence of a neurogenic bladder and the occurrence of hemorrhagic cystitis has not completely been elucidated. Therefore, further studies will be helpful to understand this association and consequently how to treat hemorrhagic cystitis.

## Conclusion

We report a rare and critical case of recurrent hemorrhagic shocks resulting from hemorrhagic cystitis.

## Funding

The authors did not receive a specific grant for this study. No part of this report has been copyrighted or published previously, and is not under consideration for publication elsewhere. Written informed consent has been obtained from the patient for this study. It has also been approved by the Institutional Reviewer Board, and the approval number is SKEC‐21‐03.

## Conflict of interest

The authors declare no conflict of interest.

## Approval of the research protocol by an institutional reviewer board

Not applicable.

## Informed consent

Not applicable.

## Registry and the registration no. of the study/trial

Not applicable.
